# Insects as Novel Ruminant Feed and a Potential Mitigation Strategy for Methane Emissions

**DOI:** 10.3390/ani11092648

**Published:** 2021-09-09

**Authors:** Eslam Ahmed, Naoki Fukuma, Masaaki Hanada, Takehiro Nishida

**Affiliations:** 1 Graduate School of Animal Husbandry, Obihiro University of Agriculture and Veterinary Medicine, Inada, Obihiro 080-8555, Japan; eslam_kh@vet.svu.edu.eg; 2 Department of Animal Behavior and Management, Faculty of Veterinary Medicine, South Valley University, Qena 83523, Egypt; 3 Department of Life and Food Sciences, Obihiro University of Agriculture and Veterinary Medicine, Inada, Obihiro 080-8555, Japan; n.fukumax@obihiro.ac.jp (N.F.); hanada@obihiro.ac.jp (M.H.); 4 Research Center for Global Agromedicine, Obihiro University of Agriculture and Veterinary Medicine, Inada, Obihiro 080-8555, Japan

**Keywords:** alternative protein sources, fatty acids, insects, methane, rumen fermentation, soybean, sustainability

## Abstract

**Simple Summary:**

In the last decade, there has been a growing interest in using edible insects as animal feed due to their high nutritive value and environmental advantages over the conventional livestock feeds. Insects have been used in the diets of some animals (poultry, fish, and swine) however, their evaluation in ruminants is still limited. The current in vitro study evaluated the usage of four different kinds of edible insects to partially substitute soybean meal as an example to the conventional high-quality expensive protein sources in ruminants’ diets. This study showed that the evaluated insects had high protein and fat contents. Substitution of 25% of soybean meal with the tested insects in a ruminant diet had no adverse effect on rumen fermentation profile or nutrient digestibility. Moreover, the inclusion of some species in the diet led to a reduction of the methane production up to 16–18% which is an additional environmental benefit. The findings of this study are encouraging for further work in this promising area to improve the sustainability of livestock industry.

**Abstract:**

This study is the first to evaluate the chemical composition and impacts of four different edible insects, *Acheta domesticus* (A.d), *Brachytrupes portentosus* (B.p), *Gryllus bimaculatus* (G.b), and *Bombyx mori* (B.m), on the digestibility, rumen fermentation, and methane production when used as a substitute for 25% of the soybean meal (SBM) in a ruminant diet through in vitro incubation. The dietary treatments were 100% grass hay, 60% grass hay + 40% SBM, 60% grass hay + 30% SBM + 10% A.d, 60% grass hay + 30% SBM + 10% B.p, 60% grass hay + 30% SBM + 10% G.b, and 60% grass hay + 30% SBM + 10% B.m. The experiment was conducted as a short-term batch culture for 24 h at 39 °C, and the incubation was repeated in 3 consecutive runs. Chemical analysis of the insects showed that they were rich in fat (14–26%) with a high proportion of unsaturated fatty acids (60–70%). Additionally, the insects were rich in protein (48–61%) containing all essential amino acids and the amino acid profiles of the insects were almost the same as that of SBM. The inclusion of insects did not affect nutrient digestibility or the production of volatile fatty acids but did increase the production of ammonia-nitrogen. The addition of G.b and B.m led to decrease in methane production by up to 18% and 16%, respectively. These results reveal that substitution of 25% SBM in the diet with the tested insects had no negative impacts, and their potential to reduce methane production is an environmental benefit.

## 1. Introduction

Global food production systems are facing the great challenge of responding to the dramatic increase in the human population and meeting the growing demand for food [1]. According to the United Nations, the world will be home to approximately 9 billion people by 2050; therefore, the demand for meat and milk is expected to increase to levels that are 58% and 70% higher than those in 2010, respectively [2]. Although livestock, especially ruminants, are one of the main important sources of animal products, this sector is responsible for approximately 14–18% of anthropogenic greenhouse gases (GHG), such as methane (CH_4_) and carbon dioxide (CO_2_) [3]. Moreover, the livestock industry is considered a hungry resource; it occupies approximately 70% of the agricultural land and consumes 8% of the world’s water [4]. Therefore, any increase in animal products is severely challenged by land degradation and GHG emissions [5]. Additionally, feed cost is one of the major constraints for further development in the livestock production industry. The cost of feed is approximately 70% of the total budget, and the required protein amounts account for over 15% of the total feed cost [6]. Typically, soybean meals are the major protein source and are commonly used in ruminant diets due to their high contents of protein and essential amino acids [7]. However, soybean production is also associated with high environmental impacts [8], and its price has been increasing with some fluctuations [9]. Therefore, to meet the increasing demand for animal products in the near future, innovative solutions and alternative sustainable ingredients to replace the conventional protein in animal diets with a reduced impact on the environment are urgently required.

In recent years, the use of edible insects to substitute or reduce other expensive high-quality feed in animal diets seems to be one of the most promising solutions to the above-mentioned problems [10,11]. The production of insect biomass as animal feed has major environmental advantages over conventional sources, as insects are able to efficiently convert low-value organic by-product wastes from fruits and vegetables into sellable nutrient sources (high feed conversion efficiency), grow rapidly, require less land and water, and produce lower GHG emissions [12,13]. Moreover, insects are characterized by their high protein (42–63%) and fat (10–40%) contents, making them ideal candidates for animal diets [10,14]. Additionally, the use of insects as animal feed is likely to be more widely accepted, as it could provide a valuable opportunity to develop a novel product [15,16]. Research interest in this field is still in its infancy; however, in the last few years, there has been increasing interest in both the economic sector and scientific community [17,18].

Although no data are currently available on the rearing of insects on a commercial scale, the commercial farming of insects such as crickets for the feed market is developing in many countries, and it is projected that insect meal production will increase to 1.2 million tons by 2025 [11]. During the last five years, the number of articles detailing the usage of edible insects in animal diets has substantially increased [12]. Several published papers have shown that insects could be used as a feed ingredient to partially or completely replace soybean meal and fishmeal, such as in broiler chicken [19], laying hen [20], free-range chickens [21], quail [22], rabbit [23], swine [24], as well as carnivorous [25] and omnivorous fish [26] diets. However, evaluation of their utilization in ruminant diets is still limited to date which is related to the potential risk of mad cow disease (Bovine Spongiform Encephalopathy). The European Union regulations prohibit the use of processed animal protein to feed food-producing animals [27]. The European Union allows the usage of insect as a feed in aquaculture since July 2017, with a recent approval from the European parliament and Council, and the Standing Committee on Plants, Animals, Food and Feed in April 2021 for the usage of insects to feed poultry and pig [28]. The prohibition of insect usage as ruminant feed is also currently applied in most of the developed countries (USA, Canada, China, and Japan). In contrast, many countries, including the developing ones, have less clear or no specific laws for the usage of insects as ruminant feed [15]. Legal rules on the use of insects as feed vary across the world, but there is noticeable interest among researchers and feed producers all over the world for further innovation and research in that promising area. This would lead to changes in the countries’ regulations to allow the use of insects as ruminant feed in the near future. Importantly, prior to establish a new livestock industry based on insects as feed, the safety of insects and the substrate on which they are reared should be carefully considered.

To the best of our knowledge, two studies have been conducted in ruminants evaluating the use of black soldier fly larvae (*Hermetia illucens*), Jamaican field crickets *(Gryllus assimilis*), and mealworms (*Tenebrio molitor*) as a substitute for soybean meals on the rumen fermentation profile, digestibility, and CH_4_ production [29,30]. These in vitro studies reported that substitution of soybean meal with the tested insects reduced CH_4_ production but had a lower nutritional value in terms of lower in vitro dry matter digestibility and production of volatile fatty acids due to the higher chitin content. Therefore, there is a need to determine the optimal inclusion levels of insects in ruminant diets to achieve favourable nutritional, economic, and environmental benefits. It has been reported that there are many insect species that may be well suited for use as feed ingredients [31,32]. Therefore, the current study was conducted to evaluate the inclusion of different kinds of edible insects, e.g., *Acheta domesticus*, *Brachytrupes portentosus*, *Gryllus bimaculatus*, and *Bombyx mori,* as partial substitutes for soybean meal. We hypothesized that the inclusion of lower levels of these insect species to partially replace soybean meal might not have adverse effects on the rumen fermentation profile. To the best of our knowledge, this is the first study reporting the comprehensive chemical analysis of these insects and evaluating their effects on the rumen fermentation characteristics, nutrient digestibility, and CH_4_ production when used as partial substitutes for soybean meal in a ruminant diet.

## 2. Materials and Methods

This study was conducted in Obihiro University of Agriculture and Veterinary Medicine, Japan during the period from January to February 2021, with an average temperature of −8 °C ± 2 and 70% humidity. The experimental procedures of this study were approved by animal care and ethics committee at the Obihiro University (approval number, 20–201). The donor animals were kept and cared for by the Field Science Center, Obihiro University.

### 2.1. Basal Diets and Insects

Kleingrass (*Panicum coloratum*) hay and soybean meal were used as the basal diet. They were ground by a mill to pass through a 1 mm sieve. The chemical composition of kleingrass hay and soybean meal is described in Table 1. Four different kinds of insects in powder form were commercially purchased (Thailand Unique Co., Udon Thani, Thailand). The edible insects used in the current study were as follows: adult house crickets (*A. domesticus*), adult giant crickets (*B. portentosus*), adult field crickets (*G. bimaculatus*), and silkworm pupae (*B. mori*). The insects were raised on commercial farms (Good Agricultural Practices certified farms) and fed a mixed diet of grains and vegetables under clean, hygienic conditions. The products were heat dried (>100 °C × 9 min) and were natural without added preservatives. More details about the insect products are available on the company website (Thailand Unique Co. Udon Thani, Thailand, https://www.thailandunique.com (accessed on 9 September 2021)).

### 2.2. Donor Animals and Rumen Fluid Collection

Two ruminally fistulated non-lactating Holstein cows approximately 7 years old with an average body weight of 894 kg were used for rumen fluid collection. The animals were raised in a free stall with rubber mat floors (Bovirex, YPTECH Co., Ltd. Tokyo, Japan) sprinkled with a moisture-absorbing spray (Kumiai Chemical Industry Co., Ltd. Tokyo, Japan). Animals had free access to clean drinking water and mineral blocks (KOEN^®^ E250 TZ, Nippon Zenyaku Kogyo Co., Fukushima, Japan). The cows were fed 30 kg corn silage/day (314 g air dry matter/kg (ADM), 937 g organic matter/kg (OM), 80 g crude protein/kg (CP), 608 g neutral detergent fibre/kg (NDF), 341 g acid detergent fibre/kg (ADF), and 76 g acid detergent lignin/kg (ADL) on a dry matter (DM) basis) supplemented with 1 kg Japanese white birch (*Betula platyphylla*)/day (ADM, 556 g/kg; OM, 994 g/kg; CP, 7 g/kg; NDF, 654 g/kg; ADF, 642 g/kg; and ADL, 178 g/kg on a DM basis) according to the maintenance level for energy. The requirements were calculated according to the guidelines of Japanese feeding standards for dairy cattle [33]. The cows were fed twice daily at 8:00 and 16:00 h. One hour after the morning feeding, 1.2 L of rumen fluid was collected from both cows at four different locations in the rumen. Then it was strained through four layers of gauze, mixed and placed into a thermos flask that had been pre-warmed at 39 °C and immediately transferred to the laboratory within 20 min. During transportation, the thermos flask was kept in an insulated container filled with warmed water at 37–39 °C.

### 2.3. Experimental Design and In Vitro Incubation Procedure

Six experimental groups with 4 replicates each were prepared by adding approximately 500 mg of substrate to pre-weighed ANKOM filter bags (F57, ANKOM Technology, Macedon, NY, USA), which were sealed and placed in 120 mL glass bottles. The experimental groups were as follows: 1- 500 mg of Kleingrass hay (100% KG); 2- 300 mg KG + 200 mg soybean meal (SBM) (60% KG + 40% SBM); 3- 300 mg KG + 150 mg SB + 50 mg *A. domesticus* (A.d) (60% KG + 30% SBM + 10% A.d); 4- 300 mg KG + 150 mg SBM + 50 mg *B. portentosus* (B.p) (60% KG + 30% SBM + 10% B.p); 5- 300 mg KG + 150 mg SBM + 50 mg *G. bimaculatus* (G.b) (60% KG + 30% SBM + 10% G.b); and 6- 300 mg KG + 150 mg SBM + 50 mg *B. mori* (B.m) (60% KG + 30% SBM + 10% B.m).

The experiment was conducted as batch culture as reported by Menke and Steingass [34]. Under continuous CO_2_ flushing, 40 mL of artificial saliva solution at pH 6.8 prepared according to McDougall [35] with 20 mL of rumen fluid was added to each 120 mL fermentation bottle. Thereafter, the bottles were flushed with CO_2_ before sealing with butyl rubber stoppers and aluminum caps (Maruemu Co., Ltd., Osaka, Japan) then incubated for 24 h at 39 °C. This batch culture procedure was repeated in three separate runs on three consecutive weeks. In each run, two blanks without substrate were included.

### 2.4. Incubation Media Sampling

After 24 h of incubation, the total gas production was estimated, and a sample of the headspace gas was collected from each bottle and stored in a vacutainer tube (BD, Becton Drive, NJ, USA), which was stored at room temperature until CH_4_ and CO_2_ determination. Afterward, the fermentation bottle caps were removed, and the pH was immediately determined (LAQUA F-72, HORIBA Scientific, Kyoto, Japan). Approximately 1 mL of the culture media was collected into Eppendorf tubes (Eppendorf AG, Hamburg, Germany) and centrifuged at 16,000× *g* and 4 °C for 5 min. The supernatant was collected and stored at −20 °C until use for volatile fatty acids (VFA) and ammonia nitrogen (NH_3_-N) estimation. The filter bags were washed with tap water until the effluent was clear. Then, the bags were dried at 60 °C for 48 h to estimate the in vitro dry matter digestibility (IVDMD). Afterwards, the bags were used for estimation of the in vitro organic matter digestibility (IVOMD), in vitro neutral detergent fibre digestibility (IVNDFD), and in vitro acid detergent fibre digestibility (IVADFD).

### 2.5. Chemical Analysis

Chemical analysis of KG, SBM, the four kinds of insect powder, and the contents in the filter bag was performed according to the AOAC [36] standard procedures. The DM percentage was determined by drying the samples in an air-forced oven at 135 °C for 2 h (method 930.15). The OM and ash were measured by placing the samples into a muffle furnace at 500 °C for 3 h (method 942.05). The ether extract was determined according to method 920.39, while nitrogen was measured according to the method of Kjeldahl (method 984.13) using an electrical heating digester and an automatic distillation apparatus (VELP Scientifica, Usmate (MB), Italy), and then the CP was estimated as nitrogen × 6.25. The NDF, ADF, and ADL were measured and expressed as inclusive residual ash using an ANKOM^200^ fibre analyser (Ankom Technology Methods 6, 5 and 8, respectively; ANKOM Technology Corp., Macedon, NY, USA). The NDF was measured using sodium sulfite without heat-stable α-amylase. The NDF and ADF analyzes of the insects chemically represented chitin with protein and chitin with amino acids, respectively. The pure chitin content was estimated according to the formula of chitin = ADF − ADL [37].

### 2.6. Amino Acid and Fatty Acid Composition Analysis

The fatty acid and amino acid profiles of the SBM and insects were analysed by Japan Food Research Laboratories, Japan. The fatty acid composition of the samples was determined by gas chromatography (7890B Agilent Technologies, Inc. Wilmington, DE, USA) equipped with flame ionization detector. The fatty acids were separated on a 30 m × 0.25 mm ID DB-23 capillary column (Agilent J&W, Santa Clara, CA, USA). Hydrogen was used as the carrier gas at an inlet pressure of 115 kPa with splitless injection at 250 °C, and the detector temperature was 250 °C. The following temperature settings were applied: the initial temperature was held at 50 °C for 1 min, increased to 170 °C at a rate of 10 °C/min, and then increased to 210 °C at a rate of 1.2 °C/min. A volume of 1 μL was injected. Peaks were identified by injection of fatty acid methyl ester mixture (SUPELCO 37-Component FAME Mix CRM47885, Sigma-Aldrich, Bellefonte, PA, USA) as a standard. The identification of each fatty acid was calculated based on the standard’s retention time and reported as percentage of the total fatty acids. The amino acid composition, except for tryptophan, histidine, phenylalanine, and leucine, was determined by an automated amino acid analyser (JLC-500/V, JEOL Ltd., Tokyo, Japan; column, LCR-6 with 4 mm × 120 mm ID, JEOL, Co. Ltd., Tokyo, Japan). Tryptophan was analysed by high-performance liquid chromatography (HPLC, LC-40D, Shimadzu, Co., Ltd., Kyoto, Japan). The column was a CAPCELL PAK C18 AQ (4.6 mm ID × 250 mm, Shiseido Co., Ltd., Kyoto, Japan) with fluorescence detection (RF-20Axs, Shimadzu, Co. Ltd., Kyoto, Japan). The mobile phase consisted of perchloric acid and methanol (80:20). The flow rate was 0.7 mL/min, and the fluorescence excitation was at 285 nm at 40 °C. Histidine, phenylalanine and leucine were analysed by HPLC (LA8080, Hitachi High-Tech Corporation, Tokyo, Japan) on a column packed with Hitachi custom ion exchange resin (4.6 mm ID × 60 mm, Hitachi High-Tech Corporation, Tokyo, Japan). The mobile phase was PF-1~4 KANTO (KANTO CHEMICAL CO., INC., Tokyo, Japan), the flow rate was 0.35 mL/min, and fluorescence excitation was at 570 nm. Each amino acid was reported as a percentage of the total amino acid composition.

### 2.7. Gas Composition Analysis

The concentrations of CH_4_ and CO_2_ were determined by injecting 1 mL of each sample into a gas chromatograph (GC-8A, Shimadzu Corp., Kyoto, Japan) using a gastight syringe (Hamilton Company, Reno, NV, USA). Further details on the GC condition were described previously [38].

### 2.8. Volatile Fatty Acids and Ammonia-Nitrogen Analysis

The concentration of VFA in the culture supernatant was determined by HPLC (Shimadzu Corp., Kyoto, Japan). Details on samples preparation and HPLC specifications were reported in details by Ahmed et al. [39]. The samples used to determine the concentration of NH_3_-N were diluted 100-fold with 0.1 M phosphate buffer (pH 5.5) and then analysed following a modified procedure of the Fujii-Okuda method [40].

### 2.9. Statistical Analysis

Data were screened for normality using PROC UNIVARIATE of SAS version 9.4 (SAS Institute Inc., Cary, NC, USA) with the Kolmogorov–Smirnov test. The homogeneity of variances was verified using Proc REG in SAS. Data were then analysed using PROC MIXED of SAS. The model included the treatments as a fixed effect, while the three runs were considered random effects. Least square means and the standard error of the mean (SEM) were calculated, and the differences in means among experimental groups were estimated by Tukey’s test. Significant differences were accepted at *p* < 0.05 with tendencies detected when the *p* value was between 0.05 and 0.10.

## 3. Results

### 3.1. Chemical Composition

The proximate analysis showed that the four kinds of insects had a higher protein percentage (61.3%, 53.3%, 56.5%, and 52.4% for A.d, B.p, G.b, and B.m, respectively) than KG hay (10.5%) and SBM (48.3%). The results for the fat content were also higher (14.6%, 22.3%, 15.8%, and 26.7% for A.d, B.p, G.b, and B.m, respectively) compared with KG hay (2.8%) and SBM (2.3%) (Table 1 and Table 2).

The fatty acid profile of the insects showed that they were rich in unsaturated fatty acids (63.3%, 60.6%, 66.7%, and 70.4% for A.d, B.p, G.b, and B.m, respectively) (Table 3). The SBM was rich in linoleic acid (52.6%), oleic acid (15.3%), and palmitic acid (15.2%). The A.d was rich in linoleic acid (36.8%), palmitic acid (25.3%), and oleic acid (25%). Similarly, B.p, was rich in linoleic acid (34.3%), palmitic acid (26.3%), and oleic acid (24.8%). The G.b was rich in linoleic acid (37.1%), oleic acid (27.9%), and palmitic acid (24.2%). Finally, B.m was rich in α-linolenic acid (31.7%), oleic acid (31.4%), and palmitic acid (22%) (Table 3). The insects contained all the essential amino acids, and the amino acid profiles of the insects were almost the same as that of SBM (Table 4).

### 3.2. Gas Production and Composition

The production of total gas, CH_4_, and CO_2_ per digestible DM (d.DM) (mL/g) in the 60% KG + 40% SBM diet was significantly higher than that in the 100% KG diet (*p* < 0.01). Substituting 25% of SBM in the diets with A.d and B.p did not affect gas production/d.DM (mL/g), but gas production was significantly lower when SBM was replaced with G.b and B.m (*p* = 0.03, Table 5). Moreover, the inclusion of G.b and B.m significantly reduced the production of CH_4_/d.DM (mL/g) (*p* < 0.05) by 18.4% and 16.3%, respectively, when compared with 60% KG + 40% SBM diet. The same effect was shown with regards to CO_2_/d.DM. In contrast, adding A.d and B.p did not show any differences in the amounts of CH_4_ and CO_2_ produced when compared with the 60% KG + 40% SBM diet. The ratio of CH_4_/CO_2_ (mL/mL) in the produced gas when G.b was used as a supplement was significantly lower (*p* = 0.004) than that in the 60% KG + 40% SBM diet (Table 5).

### 3.3. pH, In Vitro Nutrient Digestibility and Ammonia-Nitrogen Production

The inclusion of different insects significantly increased (*p* < 0.05) the pH compared with the 60% KG + 40% SBM diet. Adding SBM to KG improved the IVDMD and IVOMD (*p* < 0.05), but it did not show the same effect with regards to IVNDFD and IVADFD (*p* > 0.05, Table 6). Substituting SBM with different kinds of insects had no effect on IVDMD, IVOMD, or IVADFD when compared with the 60% KG + 40% SBM diet (*p* > 0.05), while IVNDFD was improved by the inclusion of insects in the experimental diets, especially in the case of added A.d and B.p (*p* < 0.05, Table 6). Notably, supplementation with insects in the basal diet increased the concentration of NH_3_-N (*p* < 0.01), except for when G.b was included, as this value was comparable to the 60% KG + 40% SBM (*p* = 0.98) and 100% KG (*p* = 0.36) diets (Table 6).

### 3.4. Volatile Fatty Acids Production

The 60% KG + 40% SBM diet had an improved fermentation profile in terms of the concentration of different VFA and the production of total VFA, but the A/P ratio decreased when compared with the 100% KG diet (*p* < 0.01). Substitution of 25% of the SBM in the basal diet with all the tested insects had no effect on either the concentration of different VFA or the total VFA production compared with the 60% KG + 40% SBM diet (*p* > 0.05, Table 7).

## 4. Discussion

For eco-friendly livestock production, there is a need for new sustainable nutrient sources for feed production to face the increasing consumer demand for animal products. Great research efforts are being made to find alternative feed ingredients. The use of edible insects in animal diets to substitute expensive high-quality conventional sources is one of the potential avenues to address this problem, as insects are rich in valuable nutrients (protein, fat, energy, vitamins, and minerals) [41]. There is growing research interest in this topic, which is likely to be more widely accepted by consumers [16]. Currently, insects are commonly used in the diets of livestock (poultry, rabbits, and pigs) and aquaculture species [14,42]. However, their usage in ruminant diets is still scarce, with few promising attempts [29,43,44]. The current study evaluated the use of four kinds of edible insects as substitutes for SBM in a ruminant diet (60% grass: 40% SBM), taking into account their effects on nutrient digestibility, rumen fermentation profile, and CH_4_ production.

The chemical composition analysis performed in the current study confirmed reports from previous studies that insects are rich in protein and contain all of the essential amino acids; therefore, insects are among the potential protein sources for feeding livestock [14,45]. It is important to note that the crude protein content and amino acid profile may vary depending on the species, developmental stage, and nutritional quality of the reared substrate [46,47]. Moreover, the proximate analysis and fatty acid profile of the tested insects in the current study showed that they contain substantial amounts of fat, particularly in the form of polyunsaturated fatty acids, which has also been observed in previous studies [44,48]. Apart from high protein and fat contents, the evaluated insects had high content of fibre due to the presence of chitin. Chitin is a long-chain N-acetylglucosamine polymer and the main component of their exoskeleton (8–9%) [49,50]. This fibre component is well known to be hardly digested by animals and thus may lead to lower IVDMD and IVOMD [10,51].

Previous studies have evaluated the addition of insects or their oils at different inclusion levels to ruminant diets and have shown that the insects have a lower nutritional value, as they lead to a decrease in the IVDMD and IVOMD due to chitin and high fat content, which may lead to inhibition of rumen microbes [29,30,43,44]. In contrast to the previous findings, the inclusion level of insects used in the current study as substitutes for SBM had no adverse effects on nutrient digestibility, which may be attributed to the low inclusion level (10%) used in this study. Consequently, as digestibility was not affected by the inclusion of insects, the rumen fermentation profile in terms of VFA was not affected as well by the substitution of SBM with insects. However, the pH increased slightly with the inclusion of insects in the diet, which may be attributed to the higher amount of NH_3_-N, which was reported previously when mealworm and Jamaican field crickets (*Gryllus assimilis*) were evaluated [30].

The higher NH_3_-N concentration during the incubation of SBM and insects might be related to their high degradable protein content. Diets with higher CP contents activate rumen microbes, especially proteolytic bacteria, to degrade and proteolyze the protein into NH_3_-N [52]. This nitrogen source is utilized as a precursor of amino acids and microbial protein synthesis in the rumen [53]. The formation of NH_3_-N in the rumen depends not only on the protein content but also on other important factors, such as the protein fraction and degradation rate [54]. The SBM protein is known to be highly degradable in rumen [55]. Another theory to explain this increase in NH_3_-N might be related to the lack of highly degradable carbohydrates which in turn inhibited the capability of the microorganisms to utilize the NH_3_-N for microbial protein synthesis [56]. The relatively low concentration of NH_3_-N observed in the case of G.b might be attributed to its high content of undegradable proteins, such as neutral detergent insoluble CP (NDICP) and acid detergent insoluble CP (ADICP); however, these parameters were not analysed in this study. It has been reported previously that NDICP slowly degrades and ADICP does not degrade in rumen [57]. The same finding was also observed when Jamaican field crickets were evaluated in in vitro batch culture incubation [30].

Interestingly, inclusion of insects in the diet reduced CH_4_ production, especially with G.b and B.m (18 and 16%, respectively). This reduction may be derived from the high fat content in these insects. It is well established that the addition of fat, regardless of its source, to the ruminant diet decreases CH_4_ production [58]. Several meta-analysis studies were conducted to estimate the efficacy of dietary fat to reduce CH_4_ production. Eugène et al. [59] reported a mean CH_4_ reduction of approximately 2.2% for each percentage of lipids in the ruminant diet. Beauchemin et al., [60] reported 5.6% reduction of CH_4_ per 1% addition of dietary lipids. The mode of action by which lipids reduce enteric CH_4_ might be through reduction of the fermentation of organic matter, thus decreasing the available H_2_ for methanogens and/or a direct toxic effects on methanogenic archaea and protozoa [61,62]. The theory of reducing CH_4_ by decreasing nutrient digestibility was confirmed by Jayanegara et al. [44] following the addition of 5% insect oil to high-forage and high-concentrate diets; however, this was not observed in the current study, as nutrient digestibility was not affected. Therefore, direct inhibition of methanogens might be a possible theory in this study. The insects investigated in the current study showed that they have a high content of fat, particularly in the form of unsaturated fatty acids. The greatest reduction in CH_4_ comes from unsaturated fatty acids through bio-hydrogenation, which serves as an alternative H_2_ sink [63]. Moreover, it was reported that polyunsaturated fatty acids contribute to CH_4_ reduction through a toxic effect on cellulolytic bacteria and protozoa through disruption of the cell membrane integrity [64]. The variation in lipids that reduce CH_4_ is mainly dependent on the type of fatty acids [65]. This might be confirmed with the cases of G.b and B.m, where G.b has a high content of linoleic acid, while B.m has more α-linolenic acid. Jalc et al. [66] reported a reduction in CH_4_ of up to 13.2% and 8.3% with supplementation of linoleic acid and α-linolenic acid, respectively, to a diet containing 80% lucerne and 20% barley in a Rusitec system. Additionally, palmitic acid (a saturated fatty acid), which showed higher contents in the tested insects, has shown efficacy in suppressing methanogens by increasing cell membrane permeability [67]. Therefore, in the case of G.b and B.m, a synergistic effect of unsaturated and saturated fatty acids might occur to maximize their CH_4_ reduction power. As the efficacy of lipids to reduce CH_4_ depends on the level of supplementation [59], further research must be done with higher inclusion levels of these insects to estimate their dose-dependent efficacy for more effective CH_4_ reduction. Another factor might have played a role in CH_4_ reduction in the current study could be the chitin. Through studies, chitin and chitosan (derived through chitin deacetylation) showed the ability to modulate the rumen fermentation toward less acetate and more propionate with reducing the CH_4_ production and the methanogens population [68,69,70].

## 5. Conclusions

The current study revealed that the evaluated insects were rich in fat and protein with almost the same essential amino acid profile as that found in soybean meal. Substitution of 25% of soybean meal with the four tested insects in the ruminant diet did not adversely affect the fermentation profile or nutrient digestibility. Additionally, inclusion of *Gryllus bimaculatus* and *Bombyx mori* in the diet demonstrated the potential to reduce CH_4_ production by up to 18.4% and 16.3%, respectively. Therefore, the investigated insects could be used as a sustainable source to replace 25% of the high-quality expensive protein source soybean meal without any negative effects. Further studies with increasing inclusion levels of these insects are required to investigate their impacts when used to completely replace soybean meal and as promising candidates for more effective mitigation of CH_4_ production.

## Figures and Tables

**Table 1:** Chemical composition (% in dry matter) of the basal diet used for 24-h in vitro incubation.

%	Kleingrass	Soybean Meal
Dry matter (in fresh matter)	91.69	88.16
Organic matter	88.86	92.82
Crude ash	11.14	7.18
Crude protein	10.54	48.31
Ether extract	2.75	2.33
Neutral detergent fibre	67.40	19.51
Acid detergent fibre	35.65	9.86
Acid detergent lignin	6.59	1.09

**Table 2:** Proximate analysis (% in dry matter) of the insects used for 24-h in vitro incubation.

%	*Acheta* *domesticus*	*Brachytrupes* *portentosus*	*Gryllus* *bimaculatus*	*Bombyx mori*
Dry matter (in fresh matter)	95.57	96.13	95.55	96.55
Organic matter	94.60	95.16	94.81	94.67
Crude ash	5.40	4.84	5.19	5.33
Crude protein	61.25	53.32	56.54	52.44
Ether extract	14.63	22.29	15.82	26.71
Neutral detergent fibre	39.31	40.38	37.65	40.37
Acid detergent fibre	17.29	17.34	24.21	20.72
Acid detergent lignin	2.64	4.88	3.39	10.89
Chitin	14.65	12.46	20.82	9.83

**Table 3:** Fatty acids profile (%) of soybean meal and insects.

Fatty Acid	Soybean Meal	*Acheta* *domesticus*	*Brachytrupes portentosus*	*Gryllus* *bimaculatus*	*Bombyx mori*
14:0	0.1	0.6	0.6	0.6	0.2
16:0	15.2	25.3	26.3	24.2	22.0
16:1 (*cis*-9)	0.2	0.7	0.6	0.8	1.0
17:0	0.2	0.2	0.2	0.2	0.1
18:0	4.2	8.9	11.0	7.0	6.7
18:1 (*cis*-9)	15.3	25.0	24.8	27.9	31.4
18:2 n-6 (*cis*-9,12)	52.6	36.8	34.3	37.1	6.3
18:3 n-3 (*cis*-9,12,15)	9.8	0.8	0.9	0.9	31.7
20:0	0.3	0.3	0.3	0.4	0.3
unknown	0.9	1.5	1.3	1.0	0.4

**Table 4:** Amino acids profile (%) of soybean meal and insects.

Amino Acid	Soybean Meal	*Acheta* *domesticus*	*Brachytrupes portentosus*	*Gryllus* *bimaculatus*	*Bombyx mori*
Essential amino acids					
Arginine	7.08	7.08	7.03	6.86	5.67
Lysine	6.31	6.01	6.02	5.89	6.88
Histidine	2.82	2.52	2.58	2.64	3.62
Phenylalanine	5.19	3.73	3.62	3.66	5.07
Tyrosine	3.40	5.79	5.71	5.92	6.46
Threonine	4.07	4.18	4.07	4.05	4.65
Leucine	7.86	7.87	7.93	8.03	7.50
Isoleucine	4.69	4.50	4.43	4.36	4.53
Methionine	1.40	1.76	1.71	1.61	3.10
Cysteine	1.49	1.01	0.97	0.94	1.57
Valine	5.02	6.31	6.32	6.47	5.85
Tryptophan	1.40	1.12	1.06	1.08	1.71
Non-essential amino acids					
Alanine	4.43	9.70	10.23	10.45	5.73
Glycine	4.37	6.09	6.14	6.14	5.73
Proline	5.21	6.21	6.29	6.33	4.75
Glutamic acid	18.45	11.88	12.07	11.79	11.95
Serine	5.08	5.17	4.92	5.01	4.77
Aspartic acid	11.73	9.08	8.90	8.75	10.48

**Table 5:** Effect of substituting soybean with insects on gas production and composition from 24-h in vitro incubation (*n* = 12).

Parameter	Treatments						SEM	*p*-Value
	Kleingrass	Soybean Meal	*Acheta* *domesticus*	*Brachytrupes* *portentosus*	*Gryllus* *bimaculatus*	*Bombyx mori*		
Gas production (mL)	30.17 ^c^	40.25 ^a^	40.29 ^a^	40.21 ^a^	35.08 ^bc^	36.96 ^ab^	0.71	<0.001
Gas production/DM ^1^ (mL/g)	66.30 ^c^	89.92 ^a^	88.83 ^a^	88.33 ^a^	77.17 ^bc^	81.06 ^ab^	1.59	<0.001
Gas production/d.DM ^2^ (mL/g)	171.36 ^b^	198.99 ^a^	194.82 ^a^	192.43 ^a^	177.51 ^b^	174.58 ^b^	2.12	<0.001
CO_2_ (%)	95.25 ^a^	94.06 ^c^	93.97 ^c^	93.97 ^c^	94.58 ^b^	94.36 ^bc^	0.09	<0.001
CH_4_ (%)	4.75 ^c^	5.94 ^a^	6.03 ^a^	6.03 ^a^	5.42 ^b^	5.64 ^ab^	0.09	<0.001
CO_2_ (mL)	28.72 ^c^	37.84 ^a^	37.85 ^a^	37.77 ^ab^	33.16 ^bc^	34.84 ^ab^	0.65	<0.001
CH_4_ (mL)	1.45 ^c^	2.41 ^a^	2.44 ^a^	2.44 ^a^	1.93 ^b^	2.11 ^ab^	0.07	<0.001
CH_4_/CO_2_ ratio (mL/mL)	0.050 ^d^	0.063 ^ab^	0.064 ^ab^	0.064 ^a^	0.057 ^c^	0.060 ^bc^	0.00	<0.001
CO_2_/DM (mL/g)	63.11 ^c^	84.54 ^a^	83.44 ^a^	82.97 ^ab^	72.93 ^bc^	76.42 ^ab^	1.45	<0.001
CH_4_/DM (mL/g)	3.19 ^c^	5.37 ^a^	5.39 ^a^	5.36 ^a^	4.24 ^b^	4.63 ^ab^	0.15	<0.001
CO_2_/d.DM (mL/g)	163.17 ^b^	187.12 ^a^	183.01 ^a^	180.76 ^a^	167.82 ^b^	164.64 ^b^	1.89	<0.001
CH_4_/d.DM (mL/g)	8.19 ^c^	11.87 ^a^	11.81 ^a^	11.67 ^a^	9.69 ^b^	9.94 ^b^	0.26	<0.001

^1^ DM, Dry matter. ^2^ d.DM, Digestible dry matter. SEM: Standard error of the mean. ^a, b, c, d^ Values with different superscripts in the same row are significant different (*p* < 0.05).

**Table 6:** Effect of substituting soybean with insects on pH, digestibility, and NH_3_-N from 24-h in vitro incubation (*n* = 12).

	Treatments							
Parameter	Kleingrass	Soybean Meal	*Acheta* *domesticus*	*Brachytrupes* *portentosus*	*Gryllus* *bimaculatus*	*Bombyx mori*	SEM	*p*-Value
pH	6.59 ^bc^	6.58 ^c^	6.62 ^a^	6.61 ^ab^	6.62 ^a^	6.62 ^ab^	0.01	<0.001
IVDMD ^1^ (%)	38.60 ^b^	45.14 ^a^	45.60 ^a^	45.80 ^a^	43.28 ^a^	46.22 ^a^	1.05	<0.001
IVOMD ^2^ (%)	39.27 ^b^	47.51 ^a^	46.08 ^a^	49.77 ^a^	48.15 ^a^	45.78 ^a^	1.54	0.010
IVNDFD ^3^ (%)	31.91 ^c^	34.28 ^bc^	42.99 ^a^	45.95 ^a^	41.09 ^ab^	38.54 ^abc^	1.35	0.003
IVADFD ^4^ (%)	27.50 ^ab^	28.69 ^ab^	34.76 ^ab^	30.70 ^ab^	27.26 ^b^	35.20 ^a^	0.96	0.016
NH_3_-N ^5^ (mg/dL)	9.33 ^b^	11.68 ^b^	23.89 ^a^	18.69 ^a^	13.03 ^b^	19.45 ^a^	0.84	<0.001

^1^ IVDMD: In vitro dry matter digestibility. ^2^ IVOMD: In vitro organic matter digestibility. ^3^ IVNDFD: In vitro neutral detergent fibre digestibility. ^4^ IVADFD: In vitro acid detergent fibre digestibility. ^5^ NH_3_-N: ammonia-nitrogen. SEM: Standard error of the mean. ^a, b, c^ Values with different superscripts in the same row are significant different (*p* < 0.05).

**Table 7:** Effect of substituting soybean with insects on VFA production from 24-h in vitro incubation (*n* = 12).

	Treatments							
Parameter	Kleingrass	Soybean Meal	*Acheta* *domesticus*	*Brachytrupes* *portentosus*	*Gryllus* *bimaculatus*	*Bombyx mori*	SEM	*p*-Value
Acetate (mmol/L)	78.98 ^c^	84.99 ^ab^	85.32 ^a^	85.12 ^ab^	82.47 ^b^	84.78 ^ab^	1.71	<0.001
Propionate (mmol/L)	21.04 ^b^	24.45 ^a^	24.48 ^a^	24.46 ^a^	23.49 ^a^	24.57 ^a^	0.41	<0.001
Butyrate (mmol/L)	8.08 ^c^	9.00 ^ab^	9.16 ^a^	9.16 ^a^	8.71 ^b^	8.68 ^b^	0.15	<0.001
Total VFA ^1^ (mmol/L)	108.10 ^b^	118.44 ^a^	118.96 ^a^	118.74 ^a^	114.66 ^a^	118.02 ^a^	2.22	<0.001
Acetate (mol/100 mol)	72.94 ^a^	71.64 ^b^	71.60 ^b^	71.54 ^b^	71.78 ^b^	71.70 ^b^	0.17	<0.001
Propionate (mol/100 mol)	19.53 ^b^	20.72 ^a^	20.66 ^a^	20.70 ^a^	20.59 ^a^	20.91 ^a^	0.13	<0.001
Butyrate (mol/100 mol)	7.54 ^ab^	7.65 ^a^	7.74 ^a^	7.75 ^a^	7.62 ^a^	7.39 ^b^	0.06	<0.001
A/P ^2^ ratio	3.75 ^a^	3.46 ^b^	3.48 ^b^	3.47 ^b^	3.50 ^b^	3.44 ^b^	0.03	<0.001

^1^ VFA: Volatile fatty acids. ^2^ A/P: Acetate/Propionate. SEM: Standard error of the mean. ^a, b, c^ Values with different superscripts in the same row are significant different (*p* < 0.05).

## Data Availability

The data supporting reported results in this study are available on request from the corresponding author.

## References

[1] Tilman D., Clark M. (2014). Global diets link environmental sustainability and human health. Nature.

[2] Opio C., Gerber P., Mottet A., Falcucci A., Tempio G., MacLeod M., Vellinga T., Henderson B. (2013). Greenhouse Gas Emmission from Ruminant Supply Chains. A Global Life Cycle Assessment.

[3] Gerber P.J., Steinfeld H., Henderson B., Mottet A., Opio C., Dijkman J., Falcucci A., Tempio G. (2013). Tackling Climate Change through Livestock. A Global Assessment of Emissions and Mitigation Opportunities.

[4] Steinfeld H., Gerber P., Wassenaar T.D., Castel V., Rosales M., Rosales M., de Haan C. (2006). Livestock’s Long Shadow. Environmental Issues and Options.

[5] Smith P., Gregory P.J. (2013). Climate change and sustainable food production. Proc. Nutr. Soc..

[6] Khan S., Khan R., Sultan A., Khan M., Hayat S.U., Shahid M. (2016). Evaluating the suitability of maggot meal as a partial substitute of soya bean on the productive traits, digestibility indices and organoleptic properties of broiler meat. J. Anim. Physiol. Anim. Nutr..

[7] Jolazadeh A., Dehghan-Banadaky M., Rezayazdi K. (2015). Effects of soybean meal treated with tannins extracted from pistachio hulls on performance, ruminal fermentation, blood metabolites and nutrient digestion of Holstein bulls. Anim. Feed. Sci. Technol..

[8] Halloran A., Hansen H.H., Jensen L.S., Bruun S., Halloran A., Flore R., Vantomme P., Roos N. (2018). Comparing Environmental Impacts from Insects for Feed and Food as an Alternative to Animal Production. Edible Insects in Sustainable Food Systems.

[9] FAO The Oilcrops Monthly Price and Policy Update (MPPU). http://www.fao.org/economic/est/publications/oilcrops-publications/monthly-price-and-policy-update/en/.

[10] Gasco L., Biasato I., Dabbou S., Schiavone A., Gai F. (2019). Animals Fed Insect-Based Diets: State-of-the-Art on Digestibility, Performance and Product Quality. Animals.

[11] DiGiacomo K., Leury B. (2019). Review: Insect meal: A future source of protein feed for pigs?. Animals.

[12] Van Huis A. (2021). Prospects of insects as food and feed. Org. Agric..

[13] Sorjonen J.M., Valtonen A., Hirvisalo E., Karhapää M., Lehtovaara V.J., Lindgren J., Marnila P., Mooney P., Mäki M., Siljander-Rasi H. (2019). The plant-based by-product diets for the mass-rearing of *Acheta domesticus* and *Gryllus bimaculatus*. PLoS ONE.

[14] Makkar H.P., Tran G., Heuzé V., Ankers P. (2014). State-of-the-art on use of insects as animal feed. Anim. Feed. Sci. Technol..

[15] Lähteenmäki-Uutela A., Grmelová N.H.-E.L., Deschamps M.-H., Vandenberg G.W., Ai Z., Yumei Z., Baoru Y., Nemane V. (2017). Insects as food and feed: Laws of the European Union, United States, Canada, Mexico, Australia, and China. Eur. Food Feed. Law Rev..

[16] Digiacomo K., Akit H., Leury B.J. (2019). Insects: A novel animal-feed protein source for the Australian market. Anim. Prod. Sci..

[17] Cappellozza S., Leonardi M.G., Savoldelli S., Carminati D., Rizzolo A., Cortellino G., Terova G., Moretto E., Badaile A., Concheri G. (2019). A First Attempt to Produce Proteins from Insects by Means of a Circular Economy. Animals.

[18] Apri A.D., Komalasari K. (2020). Feed and animal nutrition: Insect as animal feed. IOP Conf. Ser. Earth Environ. Sci..

[19] Benzertiha A., Kierończyk B., Rawski M., Józefiak A., Kozłowski K., Jankowski J., Józefiak D. (2019). *Tenebrio molitor* and *Zophobas morio* Full-Fat Meals in Broiler Chicken Diets: Effects on Nutrients Digestibility, Digestive Enzyme Activities, and Cecal Microbiome. Animals.

[20] Borrelli L., Coretti L., Dipineto L., Bovera F., Menna F., Chiariotti L., Nizza A., Lembo F., Fioretti V. (2017). Insect-based diet, a promising nutritional source, modulates gut microbiota composition and SCFAs production in laying hens. Sci. Rep..

[21] Dabbou S., Gasco L., Lussiana C., Brugiapaglia A., Biasato I., Renna M., Cavallarin L., Gai F., Schiavone A. (2019). Yellow mealworm (*Tenebrio molitor* L.) larvae inclusion in diets for free-range chickens: Effects on meat quality and fatty acid profile. Renew. Agric. Food Syst..

[22] Cullere M., Woods M.J., Van Emmenes L., Pieterse E., Hoffman L.C., Zotte A.D. (2019). *Hermetia illucens* Larvae Reared on Different Substrates in Broiler Quail Diets: Effect on Physicochemical and Sensory Quality of the Quail Meat. Animals.

[23] Gasco L., Dabbou S., Trocino A., Xiccato G., Capucchio M.T., Biasato I., Dezzutto D., Birolo M., Meneguz M., Schiavone A. (2019). Effect of dietary supplementation with insect fats on growth performance, digestive efficiency and health of rabbits. J. Anim. Sci. Biotechnol..

[24] Biasato I., Renna M., Gai F., Dabbou S., Meneguz M., Perona G., Martinez S., Lajusticia A.C.B., Bergagna S., Sardi L. (2019). Partially defatted black soldier fly larva meal inclusion in piglet diets: Effects on the growth performance, nutrient digestibility, blood profile, gut morphology and histological features. J. Anim. Sci. Biotechnol..

[25] Chemello G., Renna M., Caimi C., Guerreiro I., Oliva-Teles A., Enes P., Biasato I., Schiavone A., Gai F., Gasco L. (2020). Partially Defatted *Tenebrio molitor* Larva Meal in Diets for Grow-Out Rainbow Trout, *Oncorhynchus mykiss* (Walbaum): Effects on Growth Performance, Diet Digestibility and Metabolic Responses. Animals.

[26] Fontes T.V., De De Oliveira K.R.B., Almeida I.L.G., Orlando T.M.M., Rodrigues P.B., Da Costa D.V., Rosa P.V. (2019). Digestibility of Insect Meals for Nile Tilapia Fingerlings. Animals.

[27] Bessa L.W., Pieterse E., Marais J., Hoffman L.C. (2020). Why for feed and not for human consumption? The black soldier fly larvae. Compr. Rev. Food Sci. Food Saf..

[28] Byrne J. EU Authorizes Use of PAPs in Pig and Poultry Feed. https://www.feednavigator.com/Article/2021/08/17/EU-authorizes-use-of-PAPs-in-pig-and-poultry-feed.

[29] Jayanegara A., Novandri B., Yantina N., Ridla M. (2017). Use of black soldier fly larvae (*Hermetia illucens*) to substitute soybean meal in ruminant diet: An in vitro rumen fermentation study. Vet. World.

[30] Jayanegara A., Yantina N., Novandri B., Laconi E.B., Nahrowi N., Ridla M. (2017). Evaluation of some insects as potential feed ingredients for ruminants: Chemical composition, in vitro rumen fermentation and methane emissions. J. Indones. Trop. Anim. Agric..

[31] Van Huis A. (2013). Edible Insects. Future Prospects for Food and Feed Security.

[32] Jongema Y. (2017). Worldwide List of Recorded Edible Insects.

[33] MAFF (1999). Japanese Feeding Standard for Dairy Cattle.

[34] Menke K.H., Steingass H. (1988). Estimation of the energetic feed value obtained from chemical analyses and gas production using rumen fluid. Anim. Res. Dev..

[35] McDougall E.I. (1948). Studies on ruminant saliva. The composition and output of sheep’s saliva. Biochem. J..

[36] AOAC (1995). Official Methods of Analysis.

[37] Hahn T., Roth A., Febel E., Fijalkowska M., Schmitt E., Arsiwalla T., Zibek S. (2018). New methods for high-accuracy insect chitin measurement. J. Sci. Food Agric..

[38] Ahmed E., Fukuma N., Hanada M., Nishida T. (2021). The Efficacy of Plant-Based Bioactives Supplementation to Different Proportion of Concentrate Diets on Methane Production and Rumen Fermentation Characteristics In Vitro. Animals.

[39] Ahmed E., Yano R., Fujimori M., Kand D., Hanada M., Nishida T., Fukuma N. (2021). Impacts of Mootral on Methane Production, Rumen Fermentation, and Microbial Community in an in vitro Study. Front. Vet. Sci..

[40] Kawasaki K., Min X., Li X., Hasegawa E., Sakaguchi E. (2014). Transfer of blood urea nitrogen to cecal microbial nitrogen is increased by fructo-oligosaccharide feeding in guinea pigs. Anim. Sci. J..

[41] Smetana S., Schmitt E., Mathys A. (2019). Sustainable use of *Hermetia illucens* insect biomass for feed and food: Attributional and consequential life cycle assessment. Resour. Conserv. Recycl..

[42] Gasco L., Acuti G., Bani P., Zotte A.D., Danieli P.P., De Angelis A., Fortina R., Marino R., Parisi G., Piccolo G. (2020). Insect and fish by-products as sustainable alternatives to conventional animal proteins in animal nutrition. Ital. J. Anim. Sci..

[43] Astuti D., Anggraeny A., Khotijah L., Suharti S., Jayanegara A. (2019). Performance, Physiological Status, and Rumen Fermentation Profiles of Pre- and Post-Weaning Goat Kids Fed Cricket Meal as a Protein Source. Trop. Anim. Sci. J..

[44] Jayanegara A., Gustanti R., Ridwan R., Widyastuti Y. (2020). Fatty acid profiles of some insect oils and their effects on in vitro bovine rumen fermentation and methanogenesis. Ital. J. Anim. Sci..

[45] Chakravorty J., Ghosh S., Jung C., Meyer-Rochow V.B. (2014). Nutritional composition of *Chondacris rosea* and *Brachytrupes orientalis*: Two common insects used as food by tribes of Arunachal Pradesh, India. J. Asia-Pac. Entomol..

[46] Spranghers T., Ottoboni M., Klootwijk C., Ovyn A., Deboosere S., De Meulenaer B., Michiels J., Eeckhout M., De Clercq P., De Smet S. (2017). Nutritional composition of black soldier fly (*Hermetia illucens*) prepupae reared on different organic waste substrates. J. Sci. Food Agric..

[47] Meneguz M., Schiavone A., Gai F., Dama A., Lussiana C., Renna M., Gasco L. (2018). Effect of rearing substrate on growth per-formance, waste reduction efficiency and chemical composition of black soldier fly (*Hermetia illucens*) larvae. J. Sci. Food Agric..

[48] Sánchez-Muros M.-J., Barroso F., Manzano-Agugliaro F. (2014). Insect meal as renewable source of food for animal feeding: A review. J. Clean. Prod..

[49] Kroeckel S., Harjes A.-G., Roth I., Katz H., Wuertz S., Susenbeth A., Schulz C. (2012). When a turbot catches a fly: Evaluation of a pre-pupae meal of the Black Soldier Fly (*Hermetia illucens*) as fish meal substitute—Growth performance and chitin degradation in juvenile turbot (*Psetta maxima*). Aquaculture.

[50] Finke M.D. (2007). Estimate of chitin in raw whole insects. Zoo Biol..

[51] Chaudhari S.S., Arakane Y., Specht C.A., Moussian B., Boyle D.L., Park Y., Kramer K.J., Beeman R.W., Muthukrishnan S. (2011). Knickkopf protein protects and organizes chitin in the newly synthesized insect exoskeleton. Proc. Natl. Acad. Sci. USA.

[52] Owens F., Qi S., Sapienza D. (2014). Invited Review: Applied protein nutrition of ruminants—Current status and future directions. Prof. Anim. Sci..

[53] Pengpeng W., Tan Z. (2013). Ammonia Assimilation in Rumen Bacteria: A Review. Anim. Biotechnol..

[54] Jayanegara A., Dewi S.P., Laylli N., Laconi E.B., Nahrowi N., Ridla M. (2016). Determination of Cell Wall Protein from Selected Feedstuffs and its Relationship with Ruminal Protein Digestibility in Vitro. Media Peternak..

[55] Maxin G., Ouellet D., Lapierre H. (2013). Ruminal degradability of dry matter, crude protein, and amino acids in soybean meal, canola meal, corn, and wheat dried distillers grains. J. Dairy Sci..

[56] Hristov A., Ropp J. (2003). Effect of Dietary Carbohydrate Composition and Availability on Utilization of Ruminal Ammonia Nitrogen for Milk Protein Synthesis in Dairy Cows. J. Dairy Sci..

[57] Licitra G., Hernandez T.M., Van Soest P.J. (1996). Standardization of procedures for nitrogen fractionation of ruminant feeds. Anim. Feed Sci. Technol..

[58] Patra A.K. (2016). Recent Advances in Measurement and Dietary Mitigation of Enteric Methane Emissions in Ruminants. Front. Vet. Sci..

[59] Eugène M., Massé D., Chiquette J., Benchaar C. (2008). Meta-analysis on the effects of lipid supplementation on methane production in lactating dairy cows. Can. J. Anim. Sci..

[60] Beauchemin K.A., Kreuzer M., O’Mara F., McAllister T.A. (2008). Nutritional management for enteric methane abatement: A review. Aust. J. Exp. Agric..

[61] Patra A.K. (2013). The effect of dietary fats on methane emissions, and its other effects on digestibility, rumen fermentation and lactation performance in cattle: A meta-analysis. Livest. Sci..

[62] Haque M.N. (2018). Dietary manipulation: A sustainable way to mitigate methane emissions from ruminants. J. Anim. Sci. Technol..

[63] Honan M., Feng X., Tricarico J., Kebreab E. (2021). Feed additives as a strategic approach to reduce enteric methane production in cattle: Modes of action, effectiveness and safety. Anim. Prod. Sci..

[64] Martin C., Morgavi D., Doreau M. (2010). Methane mitigation in ruminants: From microbe to the farm scale. Animals.

[65] Lopes J., Harper M., Giallongo F., Oh J., Smith L., Ortega-Perez A., Harper S., Melgar A., Kniffen D., Fabin R. (2017). Effect of high-oleic-acid soybeans on production performance, milk fatty acid composition, and enteric methane emission in dairy cows. J. Dairy Sci..

[66] Jalč D., Čertik M., Kundrikova K., Namestkova P. (2008). Effect of unsaturated C18 fatty acids (oleic, linoleic and α-linolenic acids) on ruminal fermentation and production of fatty acids isomers in artificial rumen. Vet. Med..

[67] Zhou X., Meile L., Kreuzer M., Zeitz J.O. (2013). The Effect of Saturated Fatty Acids on Methanogenesis and Cell Viability of *Methanobrevibacter ruminantium*. Archaea.

[68] Belanche A., Pinloche E., Preskett D., Newbold C.J. (2015). Effects and mode of action of chitosan and ivy fruit saponins on the microbiome, fermentation and methanogenesis in the rumen simulation technique. FEMS Microbiol. Ecol..

[69] Goiri I., Garcia A., Oregui L. (2009). Effect of chitosans on in vitro rumen digestion and fermentation of maize silage. Anim. Feed. Sci. Technol..

[70] Haryati R.P., Jayanegara A., Laconi E.B., Ridla M., Suptijah P. (2019). Evaluation of chitin and chitosan from insect as feed additives to mitigate ruminal methane emission. Proceedings of the International Conference on Biology and Applied Science (ICOBAS).

